# Hiding in Plain Sight: Factors Influencing the Neuroinflammatory Response to Sport-Related Concussion

**DOI:** 10.1089/neur.2021.0081

**Published:** 2022-05-05

**Authors:** Jason B. Tabor, Michael A. McCrea, Timothy B. Meier, Carolyn A. Emery, Chantel T. Debert

**Affiliations:** ^1^ Sport Injury Prevention Research Centre, Faculty of Kinesiology, University of Calgary, Calgary, Alberta, Canada.; ^2^ Hotchkiss Brain Institute, University of Calgary, Calgary, Alberta, Canada.; ^3^ Alberta Children's Hospital Research Institute, University of Calgary, Calgary, Alberta, Canada.; ^4^ Department of Neurosurgery, Medical College of Wisconsin, Milwaukee, Wisconsin, USA.; ^5^ Department of Clinical Neurosciences, University of Calgary, Calgary, Alberta, Canada.

**Keywords:** athletes, fluid biomarkers, hypothalamic-pituitary-adrenal axis, neuroinflammation, sport-related concussion

## Abstract

Sport-related concussion (SRC) is a major concern among athletes and clinicians around the world. Research into fluid biomarkers of SRC has made significant progress in understanding the complex underlying pathophysiology of concussion. However, little headway has been made toward clinically validating any biomarkers to improve the clinical management of SRC. A major obstacle toward clinical translation of any fluid biomarker is the heterogeneity of SRC overlapping with multiple physiological systems involved in pathology and recovery. Neuroinflammation post-SRC is one such system that may confound fluid biomarker data on many fronts. Neuroinflammatory processes consist of cell mediators, both within the central nervous system and the periphery, that play vital roles in regulating the response to brain injury. Further, neuroinflammation is influenced by many biopsychosocial variables present in most athletic populations. In this commentary, we propose that future fluid biomarker research should take a systems biology approach in the context of the neuroinflammatory response to SRC. We highlight how biological variables, such as age, sex, immune challenges, and hypothalamic-pituitary-adrenal (HPA)-axis responses to stress, may alter neuroinflammation. Further, we underscore the importance of accounting for health and lifestyle variables, such as diet, exercise, sleep, and pre-morbid medical factors, when measuring inflammatory markers of SRC. To successfully move toward clinical translation, fluid biomarker research should take a more holistic approach in study design and data interpretation, collecting information on hidden variables that may be influencing the neuroinflammatory response to SRC.

## Introduction

Considerable scientific progress has been made toward the discovery, optimization, and validation of fluid biomarkers of sport-related concussion (SRC). Leading candidate biomarkers include glial fibrillary acidic protein, ubiquitin-C-terminal hydrolase 1 (UCH-L1), Tau, neurofilament light (NF-L), and inflammatory cytokines (e.g., interleukin [IL]-1β, IL-6, and tumor necrosis factor [TNF]-α).^1–6^ Current SRC biomarker research strategies have focused on a handful of assays thought to be sensitive to various traumatic brain injury (TBI) pathologies. These biomarkers are then measured in their respective fluid mediums and assumed to be representative of the physiological state of the brain after injury. However, this approach ignores basic principles of systems biology, where multiple networks of physiological processes may influence levels of a particular biomarker or confound the relationship between biomarker and brain damage. For instance, ongoing secondary injury processes may alter the concentration of biomarkers that do not directly measure neuroinflammation (e.g., UCH-L1, Tau, and NF-L) over time, which are further compounded by fluctuating blood–brain barrier (BBB) permeability throughout recovery.^7,8^

The neuroinflammatory response to TBI, including SRC, is highly complex, incorporating multiple biopsychosocial variables that should serve as a contextual framework for future SRC biomarker research. This commentary aims to highlight key variables that may confound much of the research currently being done on both brain injury and neuroinflammatory markers of SRC.

## Discussion

**FIG. 1.**
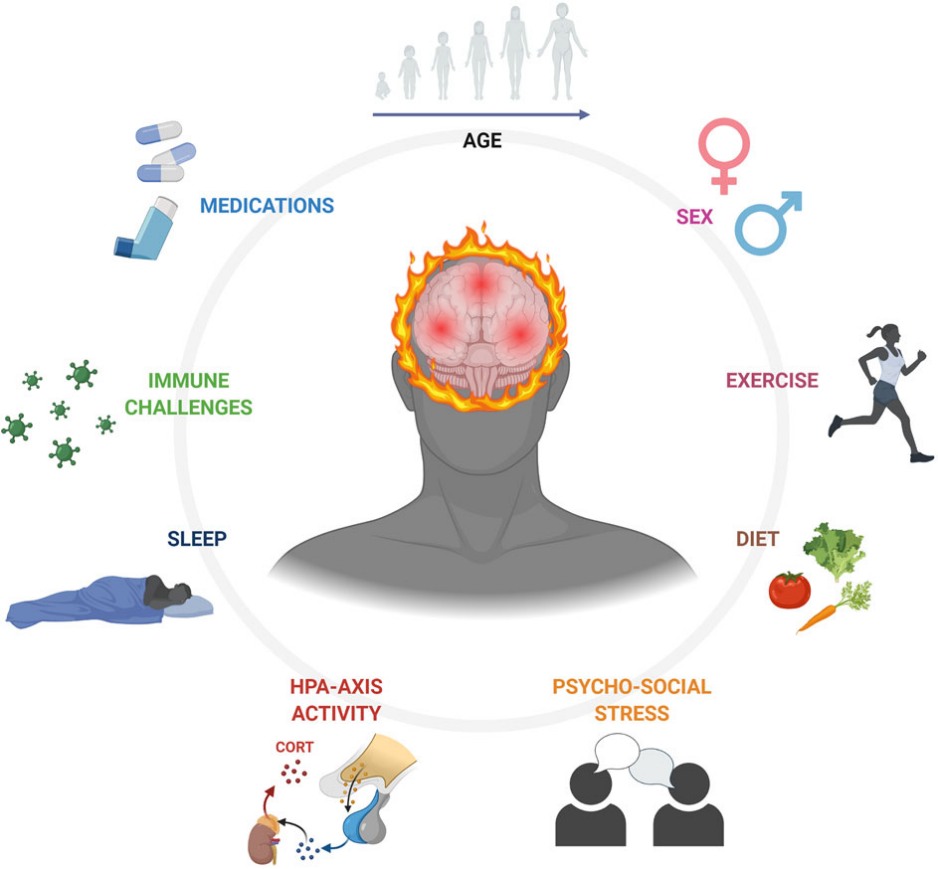
Variables influencing the neuroinflammatory response to sport-related concussion. CORT, cortisol; HPA, hypothalamic-pituitary-adrenal. Created with BioRender.com.

Many factors influence neuroinflammation (both centrally and peripherally) that make it a difficult field to study in heterogenous conditions like SRC. Age and sex are variables that must be considered for almost every aspect of SRC pathology and recovery in terms of immunological changes throughout development or sexually dimorphic inflammatory responses to injury. Moreover, many hormones exhibit regulatory functions over immunological processes. The effects of stress (physical and psychosocial) must also be considered given the bidirectional communication between the hypothalamic-pituitary-adrenal (HPA) axis and the immune system.^9^ Last, there are many lifestyle factors that can influence inflammatory processes like exercise, diet, sleep, and medications. All these confounding factors (see Fig. 1) contribute to the non-specificity of neuroinflammation and its various effects on the release and peripheral measurement of fluid biomarkers of SRC.

### Age

Age is an important variable in SRC and neuroinflammation, especially considering the physiological differences in the developing brain (e.g., ongoing synaptogenesis and myelination, higher brain water content, lower brain elasticity, and changes in cerebral blood flow and oxygen consumption)^10–12^ and anatomical vulnerabilities (e.g., larger head-to-body size ratio).^13^ Various mediators of neuroinflammation (including resident cells in the central nervous system [CNS] and infiltrating peripheral immune cells) show age-related changes that could influence measures of inflammation and subsequent markers of brain damage. For example, microglia have shown age-dependent regional functional and morphological heterogeneity in addition to more dominant immune-vigilant phenotypes in the developing brain.^14^ Peripheral immune cell composition changes throughout development, too. For instance, healthy leukocyte counts have been shown to peak in children <9 years of age, whereas neutrophil counts were highest between the ages of 15 and 18 years.^15^

In addition, reduction in monocyte chemotaxis has been reported in children compared to adults.^16^ Inflammatory responses also change throughout adulthood given that both healthy aging and age-related diseases are associated with dysregulation of nuclear factor-κB (NF-κB) pathways and cytokine networks.^17^ These age-related differences in immune function likely result in altered contributions to the neuroinflammatory response to SRC that should be considered in all biomarker studies. In the context of adolescent athletes (and depending on clinical vs non-clinical research settings), investigators may consider measuring puberty status using physical exams, hormonal measures, picture-based interviews, or self-report tools (e.g., the Pubertal Development Scale) to examine associations between neurodevelopment and biomarker outcomes.^18^

### Biological sex

Biological sex is another variable that has frequently been reported as a modifier of recovery post-TBI, but has been largely ignored in the literature concerning neuroinflammation post-SRC. Pre-clinical models are often used to study inflammatory responses to mild TBI given the ability to maintain homogenous conditions in a model organism. To that effect, there has been a major bias toward the male sex given the layer of complexity added by the female menstrual cycle. The menstrual cycle introduces variations in circulating estrogen and progesterone, which can have many anti-inflammatory effects, both centrally and peripherally.^19^ Estradiol and progesterone also interact with cells of the CNS, mediating many effects through activation of estrogen and progesterone receptors and downregulation of inflammasome activity (a proinflammatory process driven by NF-κB activation).^20–22^

In animal TBI studies, estrogen and progesterone have been shown to suppress proinflammatory cytokine production (IL-1β, IL-6, and TNFα) in microglia and overall astrogliosis, increase levels of anti-inflammatory cytokines (e.g., transforming growth factor-β and IL-10), and reduce levels of cerebral edema.^19^ In addition to female hormone cycles, testosterone levels also fluctuate in males^23^; however, the influence of testosterone has not been taken into consideration in SRC biomarker studies to date. Peripheral immune cell composition may be partially regulated by circulating hormone levels given the expression of estrogen, progesterone, and androgen receptors found on many immune cells and the sexually dimorphic responses to immune challenges (including brain injury).^24–27^ Whereas sampling sex hormone levels on all study participants would be ideal, a feasible alternative for females could be to implement self-reported menstrual cycle surveys to investigate associations between menstrual regularity and timing and the inflammatory variables in question.

### Stress, hypothalamic-pituitary-adrenal axis, and glucocorticoid activity

Physical stress in the form of injury (e.g., associated peripheral injuries or central injury as in SRC) and psychological stress (e.g., anxiety, depression) activate the HPA axis, resulting in a release of glucocorticoids (GCs; i.e., cortisol in humans) throughout the body to restore homeostasis.^28, 29^ GCs regulate inflammation with both pro- and anti-inflammatory actions depending on the context and chronicity of the stressor. In response to acute stressors, GCs display anti-inflammatory actions mediated through binding of glucocorticoid receptors (GRs), which are then translocated to the nucleus where they interact with GC responsive elements to block transcription of proinflammatory factors (e.g., downregulate NF-κB-mediated transcription of IL-1β, TNFα, and IL-6).^9^ GRs are expressed all over the body, including many cell mediators of systemic and neuroinflammation. Microglia exhibit high GR expression and are prime targets for GC-induced immunosuppression.^30^

Peripheral immune cells also express GRs, and T cells have even been reported to undergo GC-mediated apoptosis to help reduce inflammatory actions.^31^ GCs can also act on endothelial cells of the BBB to increase expression of tight junctions, thus decreasing permeability to peripheral immune cells and dampening neuroinflammation.^32,33^ In the context of SRC, potential HPA-axis dysfunction could lead to decreased GC secretion, unchecked neuroinflammation, and an impaired stress response.^9,34,35^ Additionally, animal TBI studies have shown how associated peripheral injuries may influence inflammation and recovery after brain injury, which may be relevant in clinical SRC studies.^36,37^

The opposite effect can occur where the HPA axis is excessively activated, priming microglial reactivity in the presence of excessive GC levels, resulting in maladaptive inflammation and increased neuronal death.^9^ Similar events are thought to occur as a result of psychological stressors priming and increasing microglial reactivity.^38^ Acute psychological stress has been shown to increase levels of circulating IL-1β, TNFα, and IL-6 for up to 2 h in humans.^39^ Chronic stress (i.e., sustained levels of GC in circulation) can influence systemic inflammation and the response to a traumatic event as well. For example, social determinants of health (e.g., socioeconomic status, educational and healthcare inequalities) have been associated with chronic inflammation.^40^

Studies examining the consequences of chronic stress have also shown increased monocyte mobilization from bone marrow^41^ and elevated proinflammatory cytokines in circulation of those with chronic mental health disorders like post-traumatic stress disorders or major depression.^42,43^ Given the overlap of non-specific symptoms between mental health disorders and SRC, it is reasonable to assume that psychological distress influences neuroinflammation through HPA-axis activity, which may result in altered biomarker concentrations and clinical outcomes. Considering the complex biological response to stress, researchers may benefit from evaluating cortisol levels, asking questions pertaining to various social determinants of health, and implementing psychological screening tools in their assessments.

### Lifestyle factors

Lifestyle factors that influence inflammation may be easily controlled in pre-clinical studies; however they are more difficult to account for in clinical SRC populations beyond self-reported measures. Examples of lifestyle variables include diet, exercise, and sleep quality. Many dietary variables have been shown to influence inflammation. For example, diets rich in trans-fats and high glycemic index carbohydrates lead to increased levels of proinflammatory cytokines in the general circulation.^44^ High-calorie diets have also been linked to increased neuroinflammation, thought to be one of the main drivers of various cognitive and mood disorders.^45^ Conversely, diets like the Mediterranean or ketogenic diet are associated with anti-inflammatory pathways, potentially attenuating neuroinflammation and positively influencing cognition.^46^ Alcohol and caffeine consumption have also been shown to influence both peripheral and central levels of inflammation.^47,48^ A novel, yet highly understudied, field in SRC research is beginning to discover links between nutrition, metabolism, and SRC outcomes, which might be partially attributable to changes in the gut microbiome and systemic inflammatory states.^49–52^

Given the athletic context in which SRCs occur, the influence of exercise on measures of inflammation requires careful consideration. Exercise can produce both pro- and anti-inflammatory effects that stem from localized muscle tissue and peripheral immune cells.^53^ For example, IL-6 levels are released from muscle tissue after acute exercise whereas levels of TNFα and IL-1β are actively suppressed, all of which have been recently associated with SRC.^4–6,54^ Further, exercise has been shown to suppress microglial activation and downregulate the expression of many proinflammatory cytokines in the brain, mechanistically explaining (at least in part) the neuroprotective and therapeutic effects of aerobic exercise for SRC.^55,56^ Last, exercise influences regional macrophage function in: 1) skeletal muscle during physical activity; 2) adipose tissue during energy mobilization; and 3) circulation concerning the development/progression of atherosclerosis.^53^ These influences of exercise on various immunological and cytokine networks may obscure the use of inflammatory molecules as potential diagnostic or prognostic biomarkers for SRC and need to be taken into consideration in these studies.

Sleep quality can also have a major impact on inflammation, especially given that sleep disturbance is a common symptom post-SRC.^57^ Interestingly, sleep and immune function have their own bidirectional effects. Animal and human studies have shown sleep quality to change in response to infections, injury, and neurodegenerative disorders.^58^ Sleep deprivation has also been reported to increase the expression of IL-1β, IL-6, TNFα, and C-reactive protein.^59^ A potential mechanistic explanation (particularly relevant for SRC) lies in the intimate relationship between sleep and the HPA axis given that regulation of sleep onset and completion depends on the circadian release of cortisol.^9^ Conversely, sleep disruption impacts HPA-axis function, which may lead to hyperactivity in the stress response, which as outlined above, could exacerbate inflammation. Overall, many lifestyle factors are insufficiently documented and incorporated into current biomarker analyses, which will continue to limit their clinical translation to wider athletic and general populations. Investigators may consider asking targeted questions toward recent diet habits, exercise activity, and sleep quality at the time of sample collection.

### Health-related and pre-morbid host factors

Last, there are multiple health-related factors that can influence inflammation. The use of non-steroidal anti-inflammatory drugs (e.g., ibuprofen), exogenous corticosteroids (e.g., asthma medication), and even oral hormonal contraceptives may confound inflammatory data and must therefore be considered in clinical studies.^27,60,61^ Exposure to natural allergens (e.g., seasonal pollen, pet dander) may lead to increased inflammation and activated immune cells in persons with a pre-disposition to environmental allergies.^62^ Bacterial or viral infection would also activate a host immune response. Considering the current state of the COVID-19 pandemic and recent vaccination efforts, clinical SRC studies will need to document the type and recency of vaccine administration given that this will influence cytokine levels during data collection.^63,64^

## Conclusion

Despite enormous progress over the past decade, research into fluid biomarkers of SRC remains largely in its infancy given a large focus on a handful of biomarkers at single, variable time points post-injury. Studies in general for neurological disorders (including SRC) have yet to successfully identify candidate markers of underlying pathophysiology that are associated with standard clinical measures of recovery. This remains one of the largest barriers to the clinical translation and validation of the leading candidate biomarkers of SRC. However, we may not surpass this obstacle without fully understanding the complex interaction between the neuroinflammatory response to SRC and fluid biomarker measures. Many covariates, such as age, sex, stress, and lifestyle factors, may seem trivial at first glance; however, they each introduce an abundance of hidden physiological variation in different contexts, which must be considered when designing and interpreting biomarker studies. Many questions remain around how, when, and to what degree these factors may influence aspects of neuroinflammation. Further, the methods of capturing these data should be carefully selected when assessing their impact on biological outcomes, and SRC biomarker investigators may benefit from collaboration with experts across multiple domains of inflammation. A more holistic approach, rather than reductionist approach, to studying fluid biomarkers of SRC may be more optimal in moving the field toward clinical translation given that the neuroinflammatory response to SRC is consistently shown to be greater than the sum of its parts.

## Abbreviations Used

BBB: blood–brain barrier
CNS: central nervous system
GCs: glucocorticoids
GRs: glucocorticoid receptors
HPA: hypothalamic-pituitary-adrenal
IL: interleukin
NF-κB: nuclear factor-κB
NF-L: neurofilament light
SRC: sport-related concussion
TBI: traumatic brain injury
TNF: tumor necrosis factor
UCH-L1: ubiquitin-C-terminal hydrolase 1

## References

[1] McCrea M, Broglio SP, McAllister TW, et al. Association of blood biomarkers with acute sport-related concussion in collegiate athletes: findings from the NCAA and Department of Defense CARE Consortium. JAMA Netw Open 2020;3(1):e1919771. 31977061 10.1001/jamanetworkopen.2019.19771 PMC6991302

[2] Pattinson CL, Meier TB, Guedes VA, et al. Plasma biomarker concentrations associated with return to sport following sport-related concussion in collegiate athletes—a Concussion Assessment, Research, and Education (CARE) Consortium Study. JAMA Netw Open 2020;3(8):e2013191. 32852552 10.1001/jamanetworkopen.2020.13191 PMC7453307

[3] Zetterberg H, Blennow K. Fluid biomarkers for mild traumatic brain injury and related conditions. Nat Rev Neurol 2016;12(10):563–574. 10.1038/nrneurol.2016.127 27632903

[4] Meier TB, Huber DL, Bohorquez-Montoya L, et al. A prospective study of acute blood-based biomarkers for sport-related concussion. Ann Neurol 2020;87(6):907–920. 10.1002/ana.25725 PMC7477798 32215965

[5] Nitta ME, Savitz J, Nelson LD, et al. Acute elevation of serum inflammatory markers predicts symptom recovery after concussion. Neurology 2019;93(5):e497–e507. 10.1212/WNL.0000000000007864 PMC6693429 31270219

[6] Mc Fie S, Abrahams S, Patricios J, et al. Inflammatory and apoptotic signalling pathways and concussion severity: a genetic association study. J Sports Sci 2018;36(19):2226–2234. 10.1080/02640414.2018.1448570 29509495

[7] Cash A, Theus MH. Mechanisms of blood-brain barrier dysfunction in traumatic brain injury. Int J Mol Sci 2020;21(9):3344. 10.3390/ijms21093344 PMC7246537 32397302

[8] Thelin EP, Zeiler FA, Ercole A, et al. Serial sampling of serum protein biomarkers for monitoring human traumatic brain injury dynamics: a systematic review. Front Neurol 2017;8:300. 28717351 10.3389/fneur.2017.00300 PMC5494601

[9] Kokiko-Cochran ON, Godbout JP, Tapp ZM. A tilted axis: maladaptive inflammation and HPA axis dysfunction contribute to consequences of TBI. Front Neurol 2019;10:345. 31068886 10.3389/fneur.2019.00345 PMC6491704

[10] Figaji AA. Anatomical and physiological differences between children and adults relevant to traumatic brain injury and the implications for clinical assessment and care. Front Neurol 2017;8:685. 29312119 10.3389/fneur.2017.00685 PMC5735372

[11] Takahashi T, Shirane R, Sato S, et al. Developmental changes of cerebral blood flow and oxygen metabolism in children. Am J Neuroradiol 1999;20(5):917–922. PMC7056161 10369366

[12] Fraunberger E, Esser MJ. Neuro-inflammation in pediatric traumatic brain injury—from mechanisms to inflammatory networks. Brain Sci 2019;9(11):319. 10.3390/brainsci9110319 PMC6895990 31717597

[13] Prange MT, Margulies SS. Regional, directional, and age-dependent properties of the brain undergoing large deformation. J Biomech Eng 2002;124(2):244–252. 10.1115/1.1449907 12002135

[14] Grabert K, Michoel T, Karavolos MH, et al. Microglial brain region-dependent diversity and selective regional sensitivities to aging. Nat Neurosci 2016;19(3):504–516. 10.1038/nn.4222 PMC4768346 26780511

[15] Aldrimer M, Ridefelt P, Rödöö P, et al. Population-based pediatric reference intervals for hematology, iron and transferrin. Scandinavian journal of clinical and laboratory investigation, 2013;73(3):253–261. 10.3109/00365513.2013.769625 23448533

[16] Yegin O. Chemotaxis in childhood. Pediatr Res 1983;17(3):183–187. 10.1203/00006450-198303000-00002 6835722

[17] Rea IM, Gibson DS, McGilligan V, et al. Age and age-related diseases: role of inflammation triggers and cytokines. Front Immunol 2018;9:586. 29686666 10.3389/fimmu.2018.00586 PMC5900450

[18] Shirtcliff EA, Dahl RE, Pollak SD. Pubertal development: correspondence between hormonal and physical development. Child Dev 2009;80(2):327–337. 10.1111/j.1467-8624.2009.01263.x PMC2727719 19466995

[19] Yilmaz C, Karali K, Fodelianaki G, et al. Neurosteroids as regulators of neuroinflammation. Front Neuroendocrinol 2019;55:100788. 31513776 10.1016/j.yfrne.2019.100788

[20] Kelley N, Jeltema D, Duan Y, et al. The NLRP3 inflammasome: an overview of mechanisms of activation and regulation. Int J Mol Sci 2019;20(13):3328. 10.3390/ijms20133328 PMC6651423 31284572

[21] Thakkar R, Wang R, Sareddy G, et al. NLRP3 inflammasome activation in the brain after global cerebral ischemia and regulation by 17β-estradiol. Oxid Med Cell Longev 2016;2016:8309031. 27843532 10.1155/2016/8309031 PMC5097821

[22] Aryanpour R, Pasbakhsh P, Zibara K, et al. Progesterone therapy induces an M1 to M2 switch in microglia phenotype and suppresses NLRP3 inflammasome in a cuprizone-induced demyelination mouse model. Int Immunopharmacol 2017;51:131–139. 28830026 10.1016/j.intimp.2017.08.007

[23] Brambilla DJ, Matsumoto AM, Araujo AB, et al. The effect of diurnal variation on clinical measurement of serum testosterone and other sex hormone levels in men. J Clin Endocrinol Metab 2009;94(3):907–913. 10.1210/jc.2008-1902 PMC2681273 19088162

[24] Shepherd R, Cheung AS, Pang K, et al. Sexual dimorphism in innate immunity: the role of sex hormones and epigenetics. Front Immunol 2020;11:604000. 33584674 10.3389/fimmu.2020.604000 PMC7873844

[25] Kadel S, Kovats S. Sex hormones regulate innate immune cells and promote sex differences in respiratory virus infection. Front Immunol 2018;9:1653–1653. 30079065 10.3389/fimmu.2018.01653 PMC6062604

[26] Doran SJ, Ritzel RM, Glaser EP, et al. Sex differences in acute neuroinflammation after experimental traumatic brain injury are mediated by infiltrating myeloid cells. J Neurotrauma 2019;36(7):1040–1053. 10.1089/neu.2018.6019 PMC6444913 30259790

[27] Wunderle MK, Hoeger KM, Wasserman ME, et al. Menstrual phase as predictor of outcome after mild traumatic brain injury in women. J Head Trauma Rehabil 2014;29(5):E1–E8. 10.1097/HTR.0000000000000006 PMC5237582 24220566

[28] Arlt W, Stewart PM. Adrenal corticosteroid biosynthesis, metabolism, and action. Endocrinol Metab Clin North Am 2005;34(2):293–313. 10.1016/j.ecl.2005.01.002 15850843

[29] Spencer RL, Deak T. A users guide to HPA axis research. Physiol Behav 2017;178:43–65. 27871862 10.1016/j.physbeh.2016.11.014 PMC5451309

[30] Sierra A, Gottfried-Blackmore A, Milner TA, et al. Steroid hormone receptor expression and function in microglia. Glia 2008;56(6):659–674. 10.1002/glia.20644 18286612

[31] Herold MJ, McPherson KG, Reichardt HM. Glucocorticoids in T cell apoptosis and function. Cell Mol Life Sci 2006;63(1):60–72. 10.1007/s00018-005-5390-y PMC2792342 16314919

[32] Hue CD, Cho FS, Cao S, et al. Dexamethasone potentiates in vitro blood-brain barrier recovery after primary blast injury by glucocorticoid receptor-mediated upregulation of ZO-1 tight junction protein. J Cereb Blood Flow Metabol 2015;35(7):1191–1198. 10.1038/jcbfm.2015.38 PMC4640274 25757751

[33] Salvador E, Shityakov S, Förster C. Glucocorticoids and endothelial cell barrier function. Cell Tissue Res 2014;355(3):597–605. 10.1007/s00441-013-1762-z PMC3972429 24352805

[34] Ritchie E, Emery C, Debert C. Analysis of serum cortisol to predict recovery in paediatric sport-related concussion. Brain Inj 2018;32(4):523–528. 10.1080/02699052.2018.1429662 29400570

[35] Hacioglu A, Kelestimur F, Tanriverdi F. Pituitary dysfunction due to sports-related traumatic brain injury. Pituitary 2019;22(3):322–331. 10.1007/s11102-019-00937-z 30637622

[36] Shultz SR, Sun M, Wright DK, et al. Tibial fracture exacerbates traumatic brain injury outcomes and neuroinflammation in a novel mouse model of multitrauma. J Cereb Blood Flow Metab 2015;35(8):1339–1347. 10.1038/jcbfm.2015.56 PMC4528010 25853909

[37] McDonald SJ, Sun M, Agoston DV, et al. The effect of concomitant peripheral injury on traumatic brain injury pathobiology and outcome. J Neuroinflammation 2016;13(1):90. 27117191 10.1186/s12974-016-0555-1 PMC4847339

[38] Calcia MA, Bonsall DR, Bloomfield PS, et al. Stress and neuroinflammation: a systematic review of the effects of stress on microglia and the implications for mental illness. Psychopharmacology 2016;233(9):1637–1650. 10.1007/s00213-016-4218-9 PMC4828495 26847047

[39] Marsland AL, Walsh C, Lockwood K, et al. The effects of acute psychological stress on circulating and stimulated inflammatory markers: a systematic review and meta-analysis. Brain Behav Immun 2017;64:208–219. 28089638 10.1016/j.bbi.2017.01.011 PMC5553449

[40] Berger E, Castagné R, Chadeau-Hyam M, et al. Multi-cohort study identifies social determinants of systemic inflammation over the life course. Nat Commun 2019;10(1):773. 30770820 10.1038/s41467-019-08732-x PMC6377676

[41] Niraula A, Wang Y, Godbout JP, et al. Corticosterone production during repeated social defeat causes monocyte mobilization from the bone marrow, glucocorticoid resistance, and neurovascular adhesion molecule expression. J Neurosci 2018;38(9):2328–2340. 10.1523/JNEUROSCI.2568-17.2018 PMC5830519 29382712

[42] Tursich M, R.W.J. Neufeld RWJ, P.A. Frewen PA, et al. Association of trauma exposure with proinflammatory activity: a transdiagnostic meta-analysis. Transl Psychiatry 2014;4:e413–e413. 25050993 10.1038/tp.2014.56 PMC4119223

[43] Dowlati Y, Herrmann N, Swardfager W, et al. A meta-analysis of cytokines in major depression. Biol Psychiatry 2010;67(5):446–457. 10.1016/j.biopsych.2009.09.033 20015486

[44] Galland L. Diet and inflammation. Nutr Clin Pract 2010;25(6):634–640. 10.1177/0884533610385703 21139128

[45] Melo HM, Santos LE, Ferreira ST. Diet-derived fatty acids, brain inflammation, and mental health. Front Neurosci 2019;13:265. 30983955 10.3389/fnins.2019.00265 PMC6448040

[46] McGrattan AM, McGuinness B, McKinley MC, et al. Diet and inflammation in cognitive ageing and Alzheimer's disease. Curr Nutr Rep 2019;8(2):53–65. 10.1007/s13668-019-0271-4 PMC6486891 30949921

[47] Madeira MH, Boia R, Ambrósio AF, et al. Having a coffee break: the impact of caffeine consumption on microglia-mediated inflammation in neurodegenerative diseases. Mediators Inflamm 2017;2017:4761081. 28250576 10.1155/2017/4761081 PMC5307009

[48] Imhof A, Froehlich M, Brenner H, et al. Effect of alcohol consumption on systemic markers of inflammation. Lancet 2001;357(9258):763–767. 10.1016/S0140-6736(00)04170-2 11253971

[49] Walton SR, Kranz S, SM alin SK, et al. Factors associated with energy expenditure and energy balance in acute sport-related concussion. J Athl Train 2021;56(8):860–868. 10.4085/359-20 PMC8359710 33150378

[50] Walton SR, Malin SK, Kranz S, et al. Whole-body metabolism, carbohydrate utilization, and caloric energy balance after sport concussion: a pilot study. Sports Health 2020;12(4):382–389. 10.1177/1941738120923869 PMC7787565 32520660

[51] Walrand S, Gaulmin R, Aubin R, et al. Nutritional factors in sport-related concussion. Neurochirurgie 2021;67(3):255–258. 10.1016/j.neuchi.2021.02.001 33582206

[52] Crowson MM, McClave SA. Does the intestinal microbiome impact athletic performance? Curr Gastroenterol Rep 2020;22(11):53. 32827270 10.1007/s11894-020-00790-2

[53] Metsios GS, Moe RH, Kitas GD. Exercise and inflammation. Best Pract Res Clin Rheumatol 2020;34(2):101504. 32249021 10.1016/j.berh.2020.101504

[54] Pedersen BK, Febbraio MA. Muscle as an endocrine organ: focus on muscle-derived interleukin-6. Physiol Rev 2008;88:1379–1406. 18923185 10.1152/physrev.90100.2007

[55] Mee-Inta O, Zhao ZW, Kuo YM. Physical exercise inhibits inflammation and microglial activation. Cells 2019;8(7):691. 10.3390/cells8070691 PMC6678635 31324021

[56] Leddy JJ, Master CL, Mannix R, et al. Early targeted heart rate aerobic exercise versus placebo stretching for sport-related concussion in adolescents: a randomised controlled trial. Lancet Child Adolesc Health 2021;5(11):792–799. 10.1016/S2352-4642(21)00267-4 34600629

[57] McCrory P, Meeuwisse W, Dvorak J, et al. Consensus statement on concussion in sport—the 5th International Conference on Concussion in Sport held in Berlin, October 2016. Br J Sports Med 2017;51(11):838–847. 10.1136/bjsports-2017-097699 28446457

[58] Rathbone ATL, Tharmaradinam S, Jiang S, et al. A review of the neuro- and systemic inflammatory responses in post concussion symptoms: Introduction of the “post-inflammatory brain syndrome” PIBS. Brain Behav Immun 2015;46:1–16. 25736063 10.1016/j.bbi.2015.02.009

[59] Mullington JM, Simpson NS, Meier-Ewert HK, et al. Sleep loss and inflammation. Best Pract Res Clin Endocrinol Metab 2010;24(5):775–784. 10.1016/j.beem.2010.08.014 PMC3548567 21112025

[60] Rainsford KD. Ibuprofen: pharmacology, efficacy and safety. Inflammopharmacology 2009;17(6):275–342. 10.1007/s10787-009-0016-x 19949916

[61] Cain DW, Cidlowski JA. Immune regulation by glucocorticoids. Nat Rev Immunol 2017;17(4):233–247. 10.1038/nri.2017.1 PMC9761406 28192415

[62] Panzner P, Malkusová I, Vachová M, et al. Bronchial inflammation in seasonal allergic rhinitis with or without asthma in relation to natural exposure to pollen allergens. Allergol Immunopathol (Madr) 2015;43(1):3–9. 10.1016/j.aller.2013.06.009 24075536

[63] Furman D, Davis MM. New approaches to understanding the immune response to vaccination and infection. Vaccine 2015;33(40):5271–5281. 10.1016/j.vaccine.2015.06.117 PMC4581990 26232539

[64] Jeyanathan M, Afkhami S, Smaill F, et al. Immunological considerations for COVID-19 vaccine strategies. Nat Rev Immunol 2020;20(10):615–632. 10.1038/s41577-020-00434-6 PMC7472682 32887954

